# Maternal Health: A Case Study of Rajasthan

**DOI:** 10.3329/jhpn.v27i2.3369

**Published:** 2009-04

**Authors:** Sharad D. Iyengar, Kirti Iyengar, Vikram Gupta

**Affiliations:** ^1^ Action Research and Training for Health, 772 Fatehpura, Udaipur 313 001, India; ^2^ Sir Ratan Tata Trust, Bombay House, Homi Mody Street, Mumbai 400001, India

**Keywords:** Delivery, Maternal health, Reproductive health, Skilled birth attendance, India

## Abstract

This case study has used the results of a review of literature to understand the persistence of poor maternal health in Rajasthan, a large state of north India, and to make some conclusions on reasons for the same. The rate of reduction in Rajasthan's maternal mortality ratio (MMR) has been slow, and it has remained at 445 per 1000 livebirths in 2003. The government system provides the bulk of maternal health services. Although the service infrastructure has improved in stages, the availability of maternal health services in rural areas remains poor because of low availability of human resources, especially midwives and clinical specialists, and their non-residence in rural areas. Various national programmes, such as the Family Planning, Child Survival and Safe Motherhood and Reproductive and Child Health (phase 1 and 2), have attempted to improve maternal health; however, they have not made the desired impact either because of an earlier emphasis on ineffective strategies, slow implementation as reflected in the poor use of available resources, or lack of effective ground-level governance, as exemplified by the widespread practice of informally charging users for free services. Thirty-two percent of women delivered in institutions in 2005-2006. A 2006 government scheme to give financial incentives for delivering in government institutions has led to substantial increase in the proportion of institutional deliveries. The availability of safe abortion services is limited, resulting in a large number of informal abortion service providers and unsafe abortions, especially in rural areas. The recent scheme of *Janani Suraksha Yojana* provides an opportunity to improve maternal and neonatal health, provided the quality issues can be adequately addressed.

## INTRODUCTION

With a maternal mortality ratio (MMR) of approximately 445 per 100,000 livebirths, the state of Rajasthan contributes significantly to India's burden of maternal deaths (1). The context of Rajashan sets the stage for this high MMR, both in terms of its terrain and the sociocultural environment of women's lives. This paper reviews the context of maternal health in Rajasthan and the development and present status of maternal health services in the state.

**Fig. 1.**
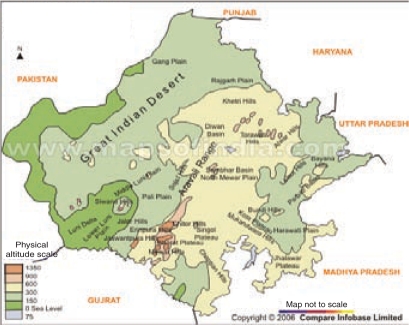
Physical map of Rajasthan

With a land area approximating 10% for India, Rajasthan is the largest state in the country. More than 60% of the state's total land area is desert, characterized by extreme temperature, low rainfall, and sparse habitation (Fig. 1). It is also the eighth most populous state of India, with a total population of 56.4 million (Census 2001), three-quarters of which lives in rural areas (Table 1) (2). The decadal growth rate continues to be high compared to other states. Over 90% of the population follows the Hindu faith, followed by 9% Muslims (3). Hindus constitute a larger proportion (95%) in the southern and south-eastern regions. Most working people in Rajasthan are engaged in agriculture and animal husbandry, although the situation in some regions is changing gradually. In areas that are better irrigated, agricultural labour is more common whereas, in the tribal-dominated south of the state, the contribution of agriculture is negligible. Under-employment is widespread, and industrial employment is low (7.5%) (4). The tribal south and the semi-arid north-central regions exhibit high rates of migration for employment; two-thirds of households in the tribal south have reported migration, with nearly half of the family income derived from sources relating to migration (5). Since 1998-1999, Rajasthan has faced regular droughts (except in 2005-2006), especially in the arid western region. With rainfall at less than 30% of the annual average, there has been severe breakdown of the livelihood support-base 6). Since women are responsible for collecting natural resources, such as water, fuel-wood, fodder, and forest-produce, droughts are known to differentially affect them. With 45 years of the last 51 years witnessing partial or total drought, a considerable amount of the state's revenue has gone into drought-relief activities. Given these factors, it is not surprising that the poverty-level in the state is high at 20.1% (4). Among its four regions, southern Rajasthan has the highest poverty-level while the western region has the lowest.

**Table 1.** Demographic profile of Rajasthan and all-India (Census 2001)

Population and demographic indicators	All-India	Rajasthan
Area (lakh sq km)	32.87	3.42
Population 2001 (million)	1,027.0	56.4
Population density (per sq km)	324	165
% rural	74.3	77.1
% urban	25.7	22.9
Literacy rate	65.4	61.0
Male literacy rate	75.8	76.5
Female literacy rate	54.2	44.3
% of scheduled castes and scheduled tribes	24.6	29.7
Sex ratio	933	922
Juvenile sex ratio [0-6 year(s) age-group]	927	909
Birth rate	26.1	31.1
Infant mortality rate	66	79
Crude death rate	8.4	7.9
Decadal growth rate	+21.3	+28.3

### Women's lives

Across caste and religious groups, a woman's personal and social status is tied to her being wife and mother. Marriage is consequently universal for girls and is governed by caste and kinship norms. Seventy-six percent of women (n=3,075) in the age-group of 20-49 years were married by the age of 18 years, according to the National Family Health Survey 3 (Table 2) (7). The literacy rate among currently married rural women was 36.2% in 2005-2006. The low family status and inadequate control by women over resources have affected many aspects of their lives. Son preference is reinforced, with women bearing more children in the quest for sons (total fertility rate in 2005-2006 was 3.2, and it was 3.6 for rural women) (7). High fertility, in turn, increases the lifetime risk of maternal death. On the other hand, in urban and some peri-urban areas, a lowering of fertility has combined with son preference in the form of sex-selective abortion. It is widely believed that this has resulted in a low juvenile [0-6 year(s)] sex ratio of 909 girls per 1,000 boys in Rajasthan (2) while the overall sex ratio is 921 females per 1,000 males (Table 1). The availability of sex-selection procedures is believed to be largely limited to district and divisional towns in the state.

**Table 2.** Marriage and fertility (NFHS 3, 2005-2006)

Indicator	Percentage
Median age	
At first marriage	15.1 years
At first cohabitation	16.5 years
% of 20-49 years old women married within 15 years of age	45.7
% of 20-49 years old women married within 18 years of age	76.0
Median age at first childbirth (25-49 years)	19.6 years
Total fertility rate	3.2

NFHS=National Family Health Survey

Women's autonomy has direct bearing on health care-seeking behaviour and healthcare-use. The National Family Health Survey (NFHS) 2005-2006 revealed that 67% of women (n=3,892) did not have access to money, and 52% of women had no say in whether they themselves could seek healthcare (7). These indicators were more adverse in rural areas. Adolescent girls are poorly nourished compared to boys. Field researchers have encountered a custom whereby families tend to underfeed pre-adolescent and adolescent girls to delay menarche and sexual maturation. Delayed sexual maturation is expected to ease the social pressure for early marriage and cohabitation. After marriage, the young bride eats last, and especially in times of drought and food scarcity, the least. These circumstances of undernutrition continue into adulthood—49% of adolescents aged 15-19 years and 37% of women aged 15-19 years have a subnormal body mass index (BMI) (7). Adolescent undernutrition is much more common in rural (39%) than in urban areas (31%), and among women belonging to the scheduled tribes (49%) than among other castes (34%). Similarly, levels of anaemia among women in the reproductive age-group (15-49 years) are 53% among ever-married women and 61% among pregnant women. Thus, undernutrition and anaemia among women continue as pervasive aspects of their adult lives.

## MATERIALS AND METHODS

To assess the present state of health services and maternal health in Rajasthan, we reviewed published and unpublished literature, including demographic and health surveys, human development reports, facility surveys, reports of non-government research organizations, such as Institute of Health Management Research (IHMR), and Action Research and Training for Health (ARTH); secondary data collected from the state health department and medical colleges (especially on health infrastructure and human resources); and reports of implementation of national programmes in the state. National and state-level demographic and health surveys, such as NFHS 1 (1992-1993), NFHS 2 (1998-1999), and NFHS 3 (2005-2006), provided information on the use of services in Rajasthan. Comparisons between these surveys provided insights into the effectiveness of various programmes, including the Child Survival and Safe Motherhood (CSSM) Programme (1992-1997) and Reproductive and Child Health (RCH) programme; and the impact of state and national population policies of 1999 and 2000 respectively. Other secondary data were drawn from facility surveys carried out by the Ministry of Health and Family Welfare, Government of India, to assess the availability and functionality of health facilities, for example, the RCH survey 1999 and 2002-2003.

## RESULTS

### Maternal mortality in Rajasthan

Information collected using different methods revealed that the MMR in Rajasthan varied from 627 per 100,000 livebirths during 1982-1986 to 445 during 2001-2003 (Table 3) (1,9,10). During all measurement periods, the MMR in Rajasthan has been higher than the national average. The lifetime risk of maternal deaths ranged from 1.9% to 2.2%, with maternal deaths being responsible for 29% of all deaths among women of reproductive age.

**Table 3.** Trends in MMR, Rajasthan

Source	MMR	MM rate	Lifetime risk (%)	Maternal deaths as a proportion of deaths of all women of reproductive age
Bhat PN *et al.* (8)	627	110	-	29
Sample Registration System 1998 (9)	670	-	-	-
Retrospective MMR survey, 1997-1998	508	64.7	2.2	-
1999-2001 SRS prospective household reports (1)	501	65.5	2.3	-
2001-2003, special survey of deaths using RHIME (1)	445	56.1	1.9	-

MM=Maternal mortality; MMR=Maternal mortality ratio; RHIME=Representative resampled, routine household interview of mortality with medical evaluation; SRS=Sample Registration System

According to the reports of the World Health Organization (WHO), the leading causes of maternal deaths in South Asia are haemorrhage (30.8%), sepsis (11.6%), anaemia (12.8%), and other indirect causes (12.5% (11). In Table 4, we have compared data on causes of death from many studies in India, including two from Rajasthan. Anaemia exerts a huge toll of women, contributing to 24% of all maternal deaths in one hospital study (12) while indirect causes, such as anaemia, tuberculosis, malaria, and heart disease, were responsible for nearly one-third of all maternal deaths, according to the Sample Registration System SRS, 1998 of the Government of India (10).

**Table 4.** Causes of maternal mortality from Indian studies (%), 1994-2003

Cause	Community-based studies			Hospital- based studies
	SRS India, 1998 (10)	SRS, Rajasthan 1998 (10)	India, EAG states, 2001-2003 special survey of deaths (1)	Pendse V, Udaipur, Rajasthan (1994-1995) (12)
Abortion	8.9	34.9	10	15
Infection/puerperal complications	16.1	4.8	11	
Haemorrhage	29.6	14.3	37	31
Obstructed labour	9.5	4.8	5	7
Eclampsia	8.3	6.3	4	13
Other direct causes				
All direct causes	72.4	65.1	67	66
Anaemia	19.0	7.9		24
Tuberculosis	4.6	15.9		
Hepatitis, heart disease	0.4	1.6		
Malaria and other indirect causes	1.4	7.9		-
Other	2.1	1.6		
All indirect causes	25.4	33.3	33	24
Other (not specified)	2.1	1.6		10

### Use of maternal health services

#### Antenatal care

By 2005-2006, three-quarters (75%) of all women who recently became pregnant (n=1,402) had received some antenatal care (ANC), a doubling since 1992 but even so, less than half of all such women had three antenatal contacts (Table 5). The majority (66%) of women started receiving ANC after the first trimester. The NFHS 3 showed that rural women were far less likely to receive three ANC contacts (32%) compared to their urban counterparts (75%). Women with 10 or more years of education were more likely to have had three antenatal care contacts (88%) compared to illiterate women (29%) (8). Government services were the major source of ANC, and nurse-midwives or other health professionals were the primary care providers (39%). The proportion of women receiving two or more tetanus injections has been increasing consistently over the last 15 years—from 29% in 1992-1993 (NFHS 1) to 65% in 2005-2006 (NFHS 3). Supplements of iron and folic acid (IFA) tablets reached 58% of women; however, only 13% consumed IFA tablets for 90 days or more (7). Although 73% of women had contacts with health professionals during pregnancy, less than half underwent essential examinations, such as blood pressure and blood test for anaemia (Table 6). Less than one-sixth of women received advice about danger-signs or place of delivery. Other surveys revealed a similar picture (13,14).

**Table 5.** Coverage (%) of antenatal care in Rajasthan, 1992-2006 (7, 8)

Indicator	Rajasthan over time		
	NFHS 1 (1992-1993)	NFHS 2 (1998-1999)	NFHS 3 (2005-2006)
Proportion receiving any ANC from a health professional	33	49	73
Urban	51	71	92
Rural	30	43	71
Mothers who had at least 3 ANC visits for birth of their last child	18.1	23.6	41.2
Mothers who consumed IFA tablets for 90 days or more	NA	NA	13.1
Percentage of women who received tetanus immunizations (2 or more injections)	29	52	65
Pregnant women, aged 15-49 years, who are anaemic	NA	51.4	61
Type of care provider			
Doctor			34
ANM/nurse/LHV or other health professional			39

ANC=Antenatal care; ANM=Auxiliary Nurse Midwife; IFA=Iron and folic acid; LHV=Lady Health Visitor; NA=Not available; NFHS=National Family Health Survey

**Table 6.** Quality of antenatal care by percentage of attending women

Indicator	NFHS 2 (1998-1999)	NFHS 3 (2005-2006)
Elements of antenatal care		
Abdomen examined	28.5	66.7
Blood pressure measured	21	44.7
Weight measured	15	45.7
Blood tested	24	45.6
Urine tested	20	42.9
% receiving information on specific pregnancy-related complications		
Vaginal bleeding	10	15.7
Convulsions	-	14.3
Prolonged labour	-	15.2
Where to go if experience pregnancy-related complications	NA	36.0
Advice about delivery care	9	NA

NA=Not available; NFHS=National Family Health Survey

### Delivery care

The proportion of women delivering in an institution rose steadily, reaching nearly one-third by 2005-2006 (NFHS 3) (Table 7). However, wide urban-rural differences remained, with nearly 70% of urban women delivering in an institution while only 23% of rural women did so. In 2005-2006, only 43% of births were attended by a health professional; urban women were more than twice as likely to seek such assistance (8). Besides residence, determinants of use of skilled attendance included younger age of women, a birth order of one, and the greater number of ANC visits. Seventy percent of women with more than four ANC visits were served by skilled attendants during childbirth. More institutional deliveries were conducted in government facilities than in private facilities, although incremental growth in deliveries in the private sector was greater. In 1998-1999, 15.9% of deliveries were in public facilities, and 5.6% were in private facilities (8) while, in 2005-2006, 19.0% and 11% of deliveries occurred in public and private facilities respectively (8). The proportion of women delivering in institutions changed rapidly following the national introduction of a maternity benefit scheme called *Janani Suraksha Yojana* (literally meaning “mothers' protection plan”) or JSY ((Fig. 3). In the second year (2006-2007) of its implementation, the number of institutional deliveries increased to 35%. The quantum of increase was the greatest in primary health centres (PHCs) and community health centres (CHCs) (99% and 57% respectively) that had conducted a few deliveries till that point (15). In 2006-2007, urban facilities witnessed a 64% increase in the number of deliveries while rural facilities saw only a 12% increase (15). The situation changed further in 2007-2008 when the number of institutional deliveries in the state crossed 1,000,000. Deliveries by caesarean section were low (3.8%) in Rajasthan but five times higher in urban (10%) than in rural areas (2.2%) (8).

**Fig. 2.**
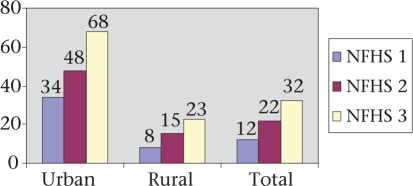
Treads in institutional delivery in Rajasthan

NFHS=National Family Health Survey

**Fig. 3.**
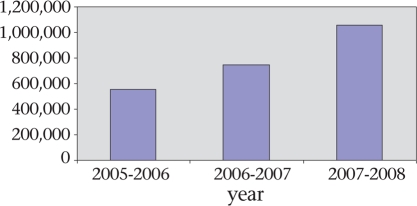
Influence of JSY on institutional delivery

JSY=*Janani Suraksha Yojana*

**Table 7.** Delivery characteristics in Rajasthan (%), 1992-2006

Characteristics	NFHS 1 (1992-1993)	NFHS 2 (1998-1999)	NFHS 3 (2005-2006)
Births assisted by doctor/nurse/LHV/ANM/other health personnel	19	36	43
Urban	-	62	77
Rural	-	29	35
Institutional births	12	22	32
Urban	34	48	68
Rural	8	15	23

ANM=Auxiliary Nurse Midwife; LHV=Lady Health Visitor; NFHS=National Family Health Survey

#### Postnatal care

Less than one-third of women received postnatal care within two days of birth (NFHS 3) (Table 8). Only 7.5% of women who delivered in the home received a postnatal check-up. Seventy-one percent of those who delivered in public-health facilities and 82% of those who delivered in private health facilities had a postnatal check-up. According to the NFHS 2, essential components of care, such as an abdominal examination or breastfeeding advice, were not provided in over half of all postnatal examinations. Evidence from a qualitative study revealed that several women were discharged very early from facilities after delivery, often within 2-3 hours, without any advice about postnatal care or initiation of breastfeeding (17). This has been corroborated by a survey in two districts of Rajasthan (reported separately in this issue of the Journal), which showed that 14% of 632 women who had an institutional vaginal delivery were discharged within six hours of delivery, and 70% before 24 hours had elapsed (18). Results of a verbal autopsy study of all 57 deaths in two rural blocks showed that five deaths had occurred among women who were discharged within 1-2 hour(s) of delivery, one from postpartum haemorrhage and four from sepsis (19). The Government of Rajasthan has recently issued guidelines that the government facilities should discharge women only after 48 hours of delivery— now a prerequisite for releasing the JSY maternity benefit.

**Table 8.** Postpartum check-ups (%) in Rajasthan, 1998-2006

Indicator	NFHS 2 (1998-1999)	NFHS 3 (2005-2006)
Mothers who received postnatal check-up within 42 days after delivery for their last childbirth	NA	31.8
Mothers who received postnatal check-up within 2 days after delivery for their last childbirth	NA	28.9
% of non-institutional births followed by a postpartum check-up within 42 days of childbirth	6.4	10.9
% of women with a postnatal check-up within 2 days after childbirth by place of delivery		
Public health facility	NA	71.2
Private health facility	NA	81.5
Home	0.5	7.5
Components of postpartum check-up		
Abdominal examination	25.2	NA
Breastfeeding advice	36.1	NA
Baby-care advice	44.9	NA

NA=Not available; NFHS=National Family Health Survey

#### Contraception

**Fig. 4.**
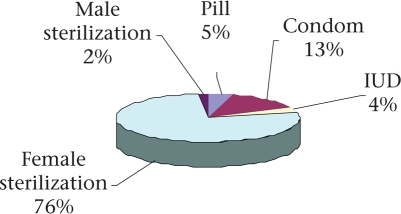
Method-mix of contraceptives in Rajasthan, 2005-2006

In 2005-2006, 47% of currently-married women in Rajasthan used a method of contraception—seven percentage points more than in 1998 (NFHS 2). The increase was greater in urban areas where 66% used contraception compared to only 41% in rural areas. Modern methods were used by 44% of women. Female sterilization was the most widely-used method, accounting for 76% of total current contraceptive-use (Fig. 4). Only 10.2% of married women used reversible contraceptive methods in 2005-2006, with the condom being the most widely-used (5.8%). Only 1.6% and 2% of women used intrauterine devices (IUDs) and oral pills respectively. Female sterilization continues to be emphasized by the government health system. In Rajasthan, even with a target-free approach, ‘expected levels of (contraceptive) achievement' (ELAs) are assigned to blocks, PHCs, and even individual health workers, and most of them (and their supervisors) consider ELAs as targets. In a qualitative study (2002) of why ANMs preferred to commute rather than reside in their work areas, several ANMs working in four blocks of Udaipur district reported that the pressure to achieve sterilization targets was a major determinant of their work routine (20).

IUD=Intrauterine device

Contraception services have largely (81% of all users) been provided by the government sector, which, however, gives much greater emphasis to terminal methods (94% of all sterilization users got it from the public sector) while pills (73% of users) and condoms (77% of users) were more often sought from private sources. The limited availability of private contraceptive provision in rural areas is reflected in the fact that reversible contraceptives were used by only 5% of rural women, against 21% in urban areas (7). With their greater emphasis on terminal methods, public family-planning services have not been able to fulfil the needs of adolescents, young women, and those with few children. This is reflected by the NFHS 3 finding that only 4.6% of women with no child and 16.5% of women with one child used modern contraception while 65% with three children used a modern contraceptive, mostly sterilization (7).

#### Abortion services

Although legally allowed for over three decades, the availability of abortion services is poor in Rajasthan. In the government sector, most CHCs and PHCs do not provide abortion services due to lack of doctors trained to carry out medical termination of pregnancy (MTP) (21). For example, in 10 districts of Rajasthan, only 39% of the CHCs and 0.5% of the PHCs provided MTP services in 2007-2008 (data collected by ARTH from offices of health authorities in 2007-2008). A few doctors managed to receive MTP training in the state—1,056 doctors were trained during 1971-2002, with an average of 35 doctors per year (22). The availability of abortion services in the private sector also is poor—a review of services has revealed that 428 certified private facilities provided abortion services in 2002 (23), or an average of 0.67 certified private facilities per 100,000 population. Not only were the number of certified private facilities low but their distribution also was skewed, with most facilities concentrating in a few districts. Nine districts with 38% of the state population had 83% of all certified facilities while the remaining 22 districts had a mere 17%. As many as five districts did not have a single certified private facility while six districts had only one each (22). The certification process for private facilities in Rajasthan is known to be tedious and time-consuming—it took an average of 14 months to get a private facility certified in 2004, with applications being returned an average of 2.4 times for resubmission (22). Eight of 19 non-certified facilities reported that they had applied but had not received any response from the authorities. In the government sector, rural PHCs/CHCs reported a very few MTPs—an average of six procedures per month while district-level government hospitals and private hospitals reported 60.5 and 49 procedures respectively per month (22). The gap between the huge demand and the low availability of abortion services has been filled by informal care providers located in villages and small towns. A study by the Indian Council of Medical Research in 1989 found a very large number of abortion providers in Rajasthan, of which 67% were from the informal sector. They included doctors from non-allopathic systems of medicine (3%), government paramedics (13%), chemists and other unqualified practitioners (14.6%), and traditional service providers (36.6%) (24). Estimates suggest that 2-10 unreported procedures are carried out for each reported one (24,25). In 2002, the most popular methods for ‘bringing on a period', i.e. terminating a possible pregnancy, were tablets of ‘EP forte' and similar drugs which, however, listed *ayurvedic* ingredients, and the injection Carboprost tromethamine, a prostaglandin (22). More recent anecdotal information suggests that the kind of ‘tablets' given by informal care providers has changed after the availability of medical abortion drugs and that misprostol has become popular.

A study of reproductive health financing in Rajasthan found that, of all women who had an abortion, 50% chose government care providers and the remaining 50% private care providers (26). While it has been claimed that women prefer using private sources for the sake of confidentiality, rural women probably opted for government services due to non-availability of private services. The mean expenditure on abortion services was Rs 1,028 (US$ 21) in 2000, an exorbitant amount for most women (Rs 903 (US$ 19) and Rs 1,500 (US$ 31) in government and private facilities respectively). The cost of abortion went up by duration of pregnancy and social vulnerability of women (out of wedlock and unmarried girls) in both private and government facilities. Follow-up of a sample of women with unwanted pregnancy visiting an interior rural health centre in southern Rajasthan, who were then referred to the city for abortion (27), revealed that only about one-sixth actually went to a facility in the city while more than half continued with their pregnancy.

### Evolving public-health service-delivery infrastructure

When Rajasthan became a state soon after independence in 1947, the total literacy rate was 9%, and there was not a single university. Medical facilities were available only in the capitals of eight erstwhile princely states, and piped water was available in five towns. Eight megawatts of power were being generated in the entire state, and major irrigation schemes were absent (28). Planned social and economic development began only after the democratic rule was established but caste divisions and social hierarchies continued to perpetuate a highly-stratified, unequal society that remained relatively unchallenged by social or religious reform movements or the pressures of industrialization.

For the first 50 years after independence, expansion of the public-health infrastructure, family planning, and child health were the main foci of India's health efforts, with maternal health being largely ignored. A three-tier health system based on population norms was established throughout India (Table 9). Rajasthan has done well in terms of facilities compared to population as per these norms.

**Fig. 5.**
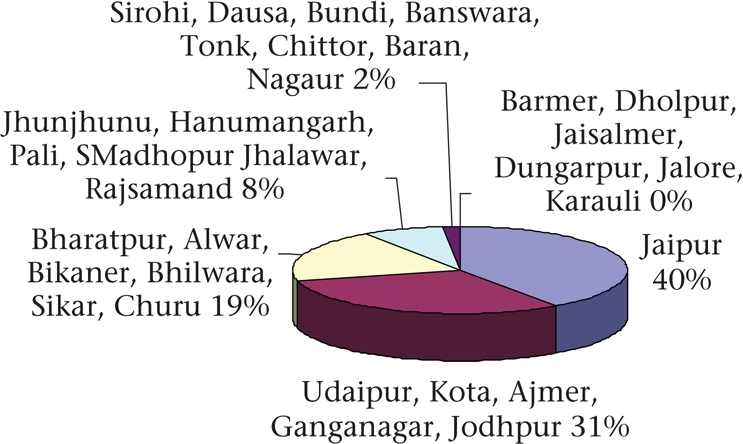
Distribution of certified private abortionfacilities in districts of Rajasthan (2002)

**Table 9.** Population norms for primary health facilities

Health facility	Staffing norms	Population coverage norms		Average radial distance (km) covered in Rajasthan	Average population coverage in Rajasthan (March 2007)
Plains area	Hilly/ tribal area
Subcentre	One female ANM	5,000	3,000	3.2	4,080
Primary Health Centre	One medical officer, one associated facility staff, supervisor	30,000	20,000	8.5	28,881
Community Health Centre	Obstetrician, surgeon, paediatrician, and specialist in medicine	120,000	80,000	17.8	128,465

ANM=Auxiliary Nurse Midwife

**Fig. 6.**
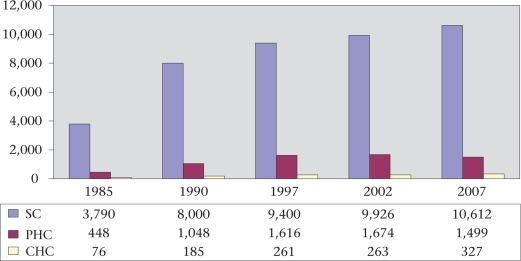
Growth of public health facilities in Rajasthan

*Number of facilities*: Rajasthan has 33 districts, 237 blocks, 9,188 *gram panchayats* (village councils), and 41,353 villages (2). There are 33 district hospitals, 144 subdivisional hospitals, 327 CHCs, 1,499 PHCs, and 10,612 SCs in the government sector (29). At the first-contact level, in addition to the SCs and PHCs, the state also has institutions following Indian systems of medicine, including 3,496 *ayurvedic* dispensaries, 92 *unani* dispensaries, and 147 homeopathic dispensaries (30). Figure 6 shows that the primary health infrastructure in the state has grown 3-4 times over the last two decades.

CHC=Community Health Centre; PHC=Primary Health Centre; SC=Subcentre

### Overall adequacy of health facilities but poor distribution

At the end of the tenth five-year plan in 2007, the state health infrastructure was nearly adequate. While there were 10% excess SCs, there was a mere 3% shortage in the number of PHCs and a 16% deficit in CHCs required as per population norms (29). However, these average values hide the non-availability of infrastructure in the tribal areas of the state where there were 46%, 52%, and 57% shortfalls in terms of of SCs, PHCs, and CHCs respectively. On 31 March 2005, the state had designated 138 facilities as First Referral Units (FRUs) (120 CHCs and 18 subdistrict hospitals). However, not all were functional as FRUs at the time.

#### Availability of human resources

Rajasthan has recently become proactive about recruiting Auxiliary Nurse Midwives (ANMs) whether at SCs or PHCs (Table 10). In 2007, there was no acute shortage of ANMs; however, 12% of posts of doctors at the PHC level were vacant (29), and the shortage of doctors was more acute in tribal areas. At the CHC level, the shortage of doctors was stark. Only 44.5% of the CHCs had a specialist, and only one-third of the CHCs had obstetricians posted in 2006-2007 (29). The situation was worse for the tribal areas where 83% of the CHCs did not have obstetricians. Although the total number of ANMs and graduate doctors is largely sufficient, their distribution is skewed—3.3% (n=352) of SCs did not have an ANM (29) in 2007. Although 13% of the PHCs oddly had four or more doctors, 9% of the PHCs did not have a single doctor. Female doctors were available only in 4.5% of the PHCs (Table 11). The availability of basic maternal health services, especially delivery and early postnatal care, is critically dependent on the availability of an ANM in her SC village. Results of a 2002 study in Udaipur district showed that 78% of the SCs had residential amenities but only 38% of ANMs (n=231) stayed in the subcentre villages (20). The study concluded that lack of supporting infrastructure and security was paramount in ensuring that the ANM stayed at her quarters (Box).

**Table 10.** Availability of human resources at CHCs, PHCs, and SCs in Rajasthan, March 2007 (29)

Human resources	Required	In position (%)	Required in tribal areas	In position in tribal areas (%)
ANM at SCs and PHCs	12,111	12,271 (101.3)	1,213	2,054 (169)
Nurse-midwives at PHCs and CHCs	3,858	8,425 (218)	414	1,483 (358)
Doctors at PHCs	1,499	1,318 (88)	162	132 (81.5)
Total specialists at CHCs	1,348	600 (44.5)	144	47 (32.7)
Obstetricians at CHCs	327	111 (34)	36	6 (17)

ANM=Auxiliary Nurse Midwife; CHC=Community Health Centre; PHC=Primary Health Centre

**Table 11.** Health facilities without staff (29)

Staff position at facilities	Total number (%)
Subcentres functioning	10,612
Subcentres without ANMs	352 (33)
Total no. of PHCs	1,499
PHCs without any doctor	130 (8.7)
PHCs with 4+ doctors	196 (13)
PHCs with a lady doctor	68 (4.5)

ANM=Auxiliary Nurse Midwife; PHC=Primary Health Centre

Box. **Reasons for ANMs' non-residence in SC villages (20)**

• Middle or high schools were available in only 24% of the SC villages
• Electrical connections were not available in 92% of the SCs, although 99% of these villages had electricity
• 44% of SC buildings had no water facility
• 51% of the SCs were located away from a main village where the ANM did not feel secure
• The ANMs faced threats and physical and sexual violence in some cases
• ANMs with rural background and those belonging to scheduled castes and tribes were more likely to stay in their field areas. As the distance of SC from a city increased, ANMs were more likely to stay in field area

ANM=Auxiliary Nurse Midwife; SC=Subcentre

#### Basic amenities, equipment, and drugs

The findings of a facility survey conducted by the Government of India in 370 districts across 26 states, including Rajasthan, in 2003 revealed that amenities, such as water, telephones, and vehicles, were severely deficient in the SCs, PHCs, CHCs, and FRUs (Table 12) (31). Adequate equipment was available in 54% of the PHCs, supplies in 69%, and adequate staff was present in 26% of the PHCs. Paramedical staff trained in CSSM was present in 33% of the PHCs. There were major gaps in facilities to provide EmOC at the CHCs and FRUs; for example, only 15% of the CHCs and 26% of the FRUs of Rajasthan had linkages with a blood-bank, and only half of the FRUs had a complete EmOC drug-kit (Table 13).

**Table 12.** Basic amenities in SCs, PHCs, CHCs, and FRUs in Rajasthan, 2003 (31)

Facility	% having facility			
	SCs	PHCs	CHCs	FRUs
Water supply	17	62	87	-
Electricity	24	80	98	97
Functional generator	NA	NA	86	67
Toilet	70	71	100	-
Labour-room	-	66	47	55
Telephone	-	5	44	63
Functional vehicle	-	9	47	55
Equipment	-	59	78	-
Supply	-	47	9	-
Medical officer staying in compound	-	32	-	-

CHCs=Community Health Centres; FRUs=First Referral Units; NA=Not available; PHCs=Primary Health Centres; SCs=Subcentres

**Table 13.** Preparedness for emergency obstetric care (%) at CHCs and FRUs (31)

Facility	CHCs	FRUs
Operation theatre	89	92
Linkage with blood-bank	15	26
Emergency obstetric care drug-kits	41	52
Obstetrician	32.5	64
Anaesthesiologist	NA	21

CHCs=Community Health Centres; FRU=First Referral Units; NA=Not available

#### Maternal health training in Rajasthan

Apart from seven government medical and affiliated nursing colleges, there are several training institutions for health personnel in Rajasthan. These include the State Institute of Health and Family Welfare (SIHFW), two Health and Family Welfare Training Centres (HFWTCs), and 27 ANM Training Centres (ANMTCs). The ANMTCs offer 18-month basic training to ANMs; 15 also conduct in-service training and have been designated as District Training Centres. The ANMTCs train about 60 ANMs each annually (in-service) and together produce approximately 1,620 new ANMs each year (32). However, the capacity for skill-based training is limited, with most providers at medical colleges already being burdened with pre-service training of undergraduates and postgraduate doctors. Besides, when training of primary care-level staff is carried out at medical colleges, they tend to observe and learn procedures and services in an over-medicalized environment that cannot be recreated in a primary-care setting (22). Such trainees also might have to compete (along a hierarchy) with in-house medical and nursing students and, hence, often do not get sufficient opportunities to learn and practice their skills. On the other hand, when primary-level institutions, such as ANM training centres, provide skill-based training (e.g. 3-week SBA training to ANMs), they face difficulties in accessing patients and providing good-quality practical training. Most ANMTCs are attached to the CHCs or district hospitals where adherence to evidence-based care and standards is deficient. For example, most facilities give routine enemas, shave the pubic area, augment labour with oxytocin (17), and give routine episiotomy for delivery while they do not use the partograph, or follow standard infection-prevention practices. Hence, students learn ‘correct' practices in the classrooms but might not get to observe them.

### The private sector in Rajasthan

**Fig. 7.**
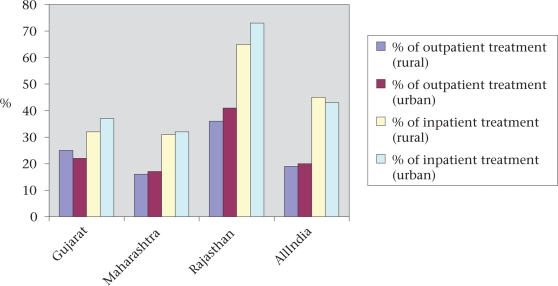
Proportion using treatment from government sources

Over the last few years, the private health sector has grown considerably in Rajasthan. A study in Jaipur city showed that bed-strength in the private sector grew 12 times during the 1960-1992 period (33). Even so, such growth was slower, when compared to states like Gujarat and Maharashtra, and to all-India levels (34). A review of the private health sector in India found 533 hospitals and nursing homes in Rajasthan in 1997 (35). The distribution of private sector facilities across districts was patchy—more than 68% of private facilities were located in six districts (Jaipur–182, Ganganagar–45, Udaipur–43, Jodhpur–39, Ajmer–32, and Kota–29) while 18 districts had less than 10 private facilities each. The rural/urban split for the private-sector health facilities is also uneven. In 2004, there were 189 government and 361 private formal facilities in Udaipur district (36). Eighty-four percent of government facilities were in rural areas while a mere 35% of formal private facilities were in rural areas. Delivery services were offered by 50% of the government facilities and 24% of the private facilities; caesarean-section facilities were available at 3% of the government facilities and 10% of the private facilities. In 1995-1996, the use of private services was lower in Rajasthan compared to other states and the national average, especially for inpatient care (Fig. 7). Thirty-five percent of inpatient care was provided through the private sector for the rural population in Rajasthan compared to 55% nationally and 69% in Maharashtra (34). With a limited formal private sector and weak public sector in rural areas, there is a large informal sector in Rajasthan that includes unqualified practitioners, practising paramedics, traditional healers, traditional birth attendants, and chemists who prescribe and dispense drugs. A survey of abortion providers in two districts in 2002-2003 found nearly 1,700 informal care providers in two districts of Rajasthan; most of them practised only on an outpatient basis. Although informal care providers have a limited role in providing delivery services, they do play an important role in providing abortion services (22).

### Use of private sector for maternal healthcare in Rajasthan

While the majority of people use private health services in India (34), use of the private sector for maternal health services has been low in Rajasthan. Only 18% of women used the private sector for antenatal care and 25% for delivery care (Table 14). The private sector was used more often for abortion services and for the treatment of reproductive health problems (Table 15).

**Table 14.** Proportion (%) using treatment from government sources (37)

Treatment from government sources	Gujarat	Maharashtra	Rajasthan	All-India
Outpatient treatment (rural)	25	16	36	19
Outpatient treatment (urban)	22	17	41	20
Hospitalized treatment (rural)	32	31	65	45
Hospitalized treatment (urban)	37	32	73	43

**Table 15.** Use of various safe motherhood services across various sectors

Indicator	Government facility	Private facility	Non-profit sector	Other, chemist, etc.
Antenatal care (outside home) (12)	81.9	18.1	0	0
Institutional delivery care (3)	74.0	25.1	0.9	0
Sterilization (3)	94.8	0.2	4.3	0.6
Reversible contraceptive methods (3)	39.4	19.8	0.3	32.7
Abortion care (14)	25	75	0	0
Treatment of reproductive health problems (3)	53.7	42.8	3.5	0

### Maternal health programmes in Rajasthan

After the International Conference on Population and Development in 1994 and the launch of India's National Reproductive and Child Health programme in 1997, maternal health began to receive the attention it deserved.

### Child Su*rvival and Safe Motherhood pr*ogramme, 1992-1997

During the first year of the eighth five-year plan (1992-1997), the Government of India, with help from the World Bank and United Nations Children's Fund (UNICEF), launched a nation wide CSSM programme with an outlay of about US$ 330 million. The CSSM programme was designed to reduce rates of infant, child and maternal mortality—the maternal health goal included reducing the MMR to 200 per 100,000 livebirths. Maternal health strategies included: (a) ensuring 100% antenatal coverage (including risk assessment during pregnancy) and tetanus immunization, (b) training of *dais* for safe delivery, (c) early detection and referral of maternal complications, and (d) setting up of First Referral Units by upgrading subdistrict hospitals and CHCs, to provide comprehensive EmOC, including caesarean section and blood transfusion. However, facilities identified as FRUs under this programme did not become fully operational mainly due to deficiency of specialist staff (obstetricians and anaesthetists). According to a facility survey conducted by the Government of India in 1999, only 16% of the CHCs in Rajasthan had an obstetrician posted and 4% had an anaesthetist. Blood-banks and blood-storage units were few and largely located at district-hospital levels—only 4% had linkage with a district blood-bank (21).

The intensified training programme of *dais* (traditional birth attendants) was part of the CSSM programme, whereby one *dai* per village was to be trained in each rural district. After six days of institutional training with some hands on practice, *dais* were supplied with safe delivery-kits. This resulted in about 20% of the existing *dais* being trained in Rajasthan by the end of 1997 (37). Findings from the NFHS 2 (1998-1999), compared with NFHS 1 (1992-1993), serve as a proxy for the impact of the CSSM programme in Rajasthan. There was some improvement in deliveries assisted by medical personnel but institutional deliveries remained low at 22% (Table 16). Despite the improvement, not even a quarter of pregnant women made three antenatal care contacts with providers, or delivered in an institution. However, an important achievement of the CSSM programme was to make high-quality useful equipment available at various service-delivery points.

**Table 16.** Impact (%) of CSSM programme, Rajasthan, 1992-1999

Indicator	NFHS 1 (1992-1993)	NFHS 2 (1998-1999)
At least 3 ANC contacts for last childbirth	18.1	23.6
Births assisted by any medical personnel	19.3	35.8
Institutional births	12	21.5

CSSM=Child Survival and Safe Motherhood; NFHS=National Family Health Survey

#### Reproductive and Child Health Programme—phase 1 (1997-2002) in Rajasthan

The Government of India launched the RCH-1 programme in 1997-1998 for five years and further extended it to March 2005. In Rajasthan, effective implementation of the programme started in 1999-2000. The essential components of the RCH 1 programme were expansion of the reproductive health service package to include reproductive tract infections (RTIs), promotion of institutional deliveries, and intensive training of *dais* in areas where the majority of deliveries occurred in the home. However, there was no emphasis on deliveries through nurse-midwives. In 1997, the target-free approach towards family planning was adopted at the national level, and nationwide targets for family planning were removed. Rajasthan, along with other parts of the country, adopted this approach. Later, the Government of Rajasthan introduced ELAs in place of targets at the district level. As mentioned earlier, at the operational level, those involved in implementing the family-planning programme have treated ELAs as targets.

First Referral Units earmarked under the CSSM programme, had not become fully operational mainly due to deficiencies of specialist staff, infrastructure, equipment, kits, and medicines. Additional staff had not been recruited. To address these problems, the RCH1 programme strengthened EmOC with more drugs and equipment and attempted to hire contractual staff (anaesthetists, obstetricians, staff nurses, ANMs, and laboratory technicians) at the FRUs. Emphasis was laid providing basic EmOC at the subdistrict level. These efforts and the implementation issues that emerged under the RCH 1 programme have been detailed in Table 17.

**Table 17.** RCH 1 programme efforts to improve maternal healthcare (as planned and Implemented) in Rajasthan

Planned	Implemented
Provision of 24-hour delivery services at PHCs/CHCs—additional honorarium for staff attending deliveries outside routine duty hours (8 pm to 7 am), in 941 CHCs and 1,178 PHCs in 32 districts (37). Provision of Rs 200 for the doctor, Rs 100 for a nurse or ANM, Rs 100 for a motivator, and Rs 30 for a cleaner for each night delivery.	By 2005-2006, 7.5% (n=129) of sampled PHCs were providing 24-hour x 7-day delivery services. Under the scheme, 78,945 ‘night' deliveries were carried out till January 2005 with an expenditure of Rs 23,775,000 (∼US$ 528,333) (37). When calculated since inception of the RCH programme in 1997 and for all 24-hour delivery centres (2,119 facilities), the number of deliveries reported works out to ∼10 deliveries per institution per year at an average cost of Rs 301 each.
Strengthening basic essential obstetric care (EOC) by increasing availability of staff at subcentres and PHCs, and by upgrading facilities.	The state contracted additional ANMs and public-health nurses in selected districts and sanctioned additional posts of doctors and other staff at the PHCs
Hiring of ANMs was considered to be an intervention to improve ANC rather than delivery care	Salaries of contractual staff were much lower than that of regular staff (e.g. contractual ANMs got Rs 3,500 and doctors got Rs 8,000), and hence, retention became a problem Facilities often did not have adequate drugs and equipment for essential obstetric care. The facility survey of 2002-2003 showed that only 36% of the PHCs had essential obstetric care drug-kit, 63% had an autoclave, and 77% had a sterilizer drum (31)
Referral transport to indigent families channeled through village councils *(panchayats)*: To improve referral of women from remote villages to a health facility in the event of a maternal complication, in selected areas of 19 districts, lump-sum assistance was made available to *panchayats* (Rs 5,000 in the first year, Rs 4,000 in the second year, Rs 3,000 in the third year, Rs 2,000 in the fourth year); under this scheme, an amount of Rs 7,205,000 (∼US$ 160,111) was released to *panchayats*	The scheme by and large remained unimplemented. By 2002, only 6.5% of funds had been used, and by January 2005, only 22% of allocated money was used at an average cost of Rs 466 per woman (37). Inability to use transport-funds stemmed from a number of issues; most importantly, families did not have information about the scheme, and even if they claimed money, payment was not released in time; and when ultimately released, it often was not the full amount
	A functional vehicle and telephone facility was present only in about half of the CHCs and FRUs (31). This severely limited the ability of these facilities to provide prompt referral
Strengthening EmOC services: Contractual staff, including anaesthetists (Rs 1,000 per case at the subdistrict, CHC and FRU levels), obstetricians, staff nurses, ANMs, and laboratory technicians to be deployed at FRUs	Hiring of anaesthetists did not commence because of a shortage of anaesthetists in the state (37). Specialist staff was missing in several FRUs—an obstetrician was present in about 64% but an anesthetist was present in only 21%, in 2003 (31)
Skill-building training of staff with preferential diploma training of doctors in anaesthesia and resuscitation for EmOC; the duration of training was doubled to two weeks	Training of doctors in anaesthesia did not start. Some doctors and nurses received training in basic EmOC as part of the AMDD project. Nine percent of medical officers received integrated (classroom) orientation-training on RCH
	However, staff nurses and ANMs were not allowed to use most life-saving drugs for maternal emergencies, nor were there clear guidelines about nurses providing delivery or emergency care in the absence of doctors, which was a common occurrence in interior facilities
Supply of safe blood to the FRUs and CHCs	Improvement in improving blood supply was insignificant. The facility survey of (2002-03) found that only 32% of the FRUs and 15% of the CHCs had linkages with district blood-banks (31)
Upgrading infrastructure and construction of operation theatres and labour-rooms in the FRUs and CHCs	Experience with infrastructure development was mixed: 74 operating theatres and 91 labour-rooms were constructed in the CHCs and district hospitals in 13 districts of the state However, the PHCs and SCs receive insufficient attention — a facility survey at the end of the RCH 1 programme revealed that an aseptic labour- room was available only in 55% (n=73) and 46% (n=89) of the FRUs and CHCs respectively. The situation of supply of equipment-kits was better with 80% of the FRUs and 87% of the CHCs having a kit for normal delivery. Telephone facility was made available only in 63% of the FRUs (31)
Introduction of financial incentives for pregnant women: The National Maternity Benefit Scheme was launched across the country during the RCH 1 programme with Rs 500 provided to ‘below poverty-line' families for delivery of the first 2 children, provided the woman's age was at least 19 years, was a resident of the state, and had registered her pregnancy with the PHC	No systematic evaluation of the NMBS was carried out. However, as of March 2004, only 8,369 women had received benefits, totaling Rs 4,184,000 (∼,977). It is estimated that this covered 3.5% of the total births expected within the BPL population of the state. One of the reasons why the scheme could not reach most women was that the eligibility requirements were complex—it was restricted to possession of a BPL card, age above 19 years, and only for the first two livebirths
Improvement of facilities for safe abortion (MTP): Ensuring that at least one team of a doctor and a nurse is trained for every district hospital and CHC	Access to safe abortion services in government facilities remained limited
Equipment for MTP in selected facilities	MTP services could not be started at the PHCs, through visiting doctors—there were no takers among doctors for the scheme
Arranging for visiting doctors (on contract) from the district to provide MTP services at the PHCs, where a regular facility is not available	A pilot project on MVA supported by the Ministry of Health and Family Welfare under the RCH programme, initiated skilled-based training. A few doctors from medical colleges of 2 districts (Jaipur and Udaipur) received training on the MVA technique. While MVA began to be used more regularly in medical colleges across the state, it did not become the preferred technique, and MTP trainees across the state got limited exposure to the same (23)
Training of traditional birth attendants	Under this scheme, 1,070 TBAs received classroom training till January 2002, with an expenditure of Rs 1,700,000. There was no assessment of change in TBAs' practices or access to maternal health services

AMDD=Averting maternal death and disability; ANC=Antenatal care; ANM=Auxiliary Nurse Midwife; BPL=Below poverty-line; CHCs=Community Health Centres; EmOC=Emergency obstetric care; FRUs=First Referral Units; MTP=Medical termination of pregnancy; MVA=Manual vacuum aspiration; NMBS=National Maternity Benefit Scheme; PHCs=Primary Health Centres; RCH=Reproductive and child health; TBA=Traditional birth attendants

### Averting Maternal Deaths and Disability Project (1999-2004)

With support from the Bill & Melinda Gates Foundation, UNICEF, and United Nations Population Fund (UNFPA), a project to increase access, quality, and use of EmOC services was implemented during 1999-2004. It was implemented through the UNFPA in seven districts (Alwar, Bharatpur, Karauli, Sawai Madhopur, Bhilwara, Chittorgarh, and Udaipur; population–13 million), and by the UNICEF in three other districts (Jhalawar, Dholpur, and Baran; population–3.2 million). A key strategy of the project was to strengthen selected CHCs and block-level PHCs to provide basic EOC (38). Additionally, comprehensive EOC services were strengthened at the district and teaching hospitals. The main interventions were: (a) training of graduate doctors to manage obstetric emergencies (12 teams of master trainers trained staff of 27 institutions), (b) procuring furniture, equipment, and essential drugs and arrangements for maintenance, (c) renovation of CHC/block PHC structures to make them client-friendly and to reduce the risk of infection, and (d) setting up blood-storage units at subdistrict hospitals.

Through this project, the availability of basic EmOC increased from 26 to 53 facilities, and the functioning of comprehensive EmOC facilities increased from 17 to 24 (39). A two-week training course on basic EmOC was designed and implemented for government doctors and nurses. There was an increase in the number of treated delivery-related complications from 5,607 in 2000 to 9,128 in 2003, indicating that more women with complicated deliveries were being referred to project institutions. Met need for EmOC increased from 8.8% to 15% (40).

### Efforts to improve maternal health under the RCH 2 programme of the National Rural Health Mission (NRHM)

In Rajasthan, the second phase of the RCH programme sponsored by the Government of India with in-built support from the UNFPA, World Bank, Department for International Development (DFID), and others started in 2005. Specific strategies of the RCH 2 programme in the state were informed by a review of the limitations and strengths of strategies adopted during the previous phase of the programme (RCH 1). Planned interventions for maternal health and their implementation are detailed in Table 18.

**Table 18.** Efforts to improve maternal health under the RCH 2 programme/NRHM in Rajasthan

Planned	Implemented
Continuation of ongoing schemes of RCH 1 programme • 24-hour delivery scheme for night delivery • Training of *dais* • Contract staff (ANMs, laboratory technicians, public-health nurses) (37) Under the NRHM, the number of ANMs per SC was to increase from one to two	The state undertook a massive recruitment drive for ANMs Additional contractual staff (public health nurse and laboratory technicians) were recruited to strengthen 50 FRUs. As of 31 March 2007, the state had 2,068 laboratory technicians against a requirement of 1,836 and 8,425 staff nurses at the PHCs and CHCs against a requirement of 3,858—a surplus of both categories (30)
Provision of basic EmOC at all CHCs Training of medical officers and other staff of CHCs in basic EmOC, ensuring that a team of 2 medical officers, one LHV, and 2 nurses provide 24-hour services at the CHCs Training of 512 doctors in basic EmOC	In 2008, 130 of 170 targeted facilities (including CHCs and PHCs) were providing basic EmOC services (30)
Increasing the number of facilities providing comprehensive EmOC Training of 200 medical officers in anesthesia at FRUs (18 weeks) • Training of 79 doctors in comprehensive EmOC • Development of blood-storage units at all FRUs, networking with district hospitals to ensure access to blood-banks • Transfer of specialist doctors to identified FRUs • Provision of imprest money to medical officers at FRUs to undertake minor repairs, etc. • Strengthening infrastructure for comprehensive EmOC and basic EmOC institutions	As of 2007-2008, 34 doctors have been trained in life-saving anaesthesia skills (30) There were 60 blood-banks in Rajasthan (41), all at the district level and at the state capital. Information on coverage of blood-storage units was not available As of 2008, apart from 33 district hospitals, which function as comprehensive EmOC centres, 15% of 337 CHCs in the state were equipped with blood-storage units (∼50 CHCs) and 18% had caesarean facilities. This takes the total number of comprehensive EmOC facilities to ∼83 (or one comprehensive EmOC facility per 750,000 people) (30)
Promotion of institutional deliveries through financial incentives: The *Janani Suraksha Yojana or* JSY scheme succeeded the NMBS. Restrictions on age of the mother, poverty status, and number of children were relaxed; hence, large numbers of women became eligible	JSY stimulated a dramatic increase in the number of institutional deliveries in Rajasthan, from 537,000, 720,000, to 1,020,000 in 2005-2006, 2006-2007, and 2007-2008 respectively. The numbers of beneficiaries of JSY payments during the same period were fewer, at 10,085, 387,648, and 774,877 respectively (42). The State Government simplified the system of making payments under JSY. Women who delivered in institutions were given a bearer cheque on discharge, which could be cashed the same day or soon thereafter
Providing skilled birth care to pregnant women, obstetric first-aid, quality ANC, and strengthening postpartum care Provision of medicines and supplies	After the Government of India revised the guidelines for ANMs as skilled birth attendants in 2005, permitting ANMs to administer life-saving drugs for dealing with maternal emergencies, Rajasthan has trained 1,236 ANMs and LHVs in skilled attendance at birth (30)
Establishment of a midwifery resource centre to provide training to trainers of ANMs for skilled birth attendance Training of ANMs to administer obstetric first-aid and use life-saving obstetric drugs	Two midwifery resource centres in Jaipur and Udaipur provided 2-week training to about 150 master trainers of SBAs, emphasizing practical skills development and evidence-based care. The SBA trainers further conducted 3-week training programmes for ANMs/staff nurses in their respective districts
Preparing PHCs (and SCs) to handle obstetric emergencies	Although several ANMs, LHVs, and staff nurses received training in skilled attendance, there are no guidelines to nurse-midwives working at the PHCs and CHCs to attend deliveries and obstetric emergencies in absence of doctors. Discussion with several nurse-midwives at the time of training courses and during monitoring field visits in 2008 revealed that delivery continued to be positioned as a doctor-based service in the majority of CHCs/PHCs. In the absence of a doctor on duty, most nurse-midwives referred women coming for deliveries to higher-level facilities. Further, only 43 (2.5%) PHCs currently had 3 staff nurses in position to provide round-the-clock services
Setting up a model SCs scheme. Although training of ANMs in skilled attendance was ongoing, not all SCs were expected to function as 24-hour delivery centres. Hence, 200 SCs were labelled as model SCs with labour-rooms	Some improvements in infrastructure and equipment occurred in model SCs but a very few had begun to provide 24-hour delivery services
Strengthening of the referral system Development of guidelines and protocols for referral services Making funds available for referral transport at the subcentre and PHC (not *panchayat*) levels Registration vouchers to pregnant women so that, if in the event of complication she went to an EmOC institution, her transport-costs would be reimbursed at the facility	The JSY allowed for reimbursement of transport-costs for reaching an institution for delivery. However, costs were not reimbursed in the event of life-threatening postpartum or pregnancy complications Guidelines and protocols for referral had not reached facilities
Promotion of safe abortion services • Provision of MVA in all comprehensive EmOC and basic EmOC facilities • Encouraging private and NGO sectors to establish quality MTP services • Promotion of use of medical abortion in public and private institutions	Implementation was slow. Only 34% of the CHCs and 0.5% of PHCs provided MTPs in 2007-2008 in Rajasthan. The number of certified facilities and trained MTP providers per 100,000 population in the state was a mere 1.2 and 1.7 in 2007-2008, with most facilities and providers being concentrated in urban areas (23)
	Till 2008, medical abortion drugs were not made available through government supplies. To prevent over-the-counter misuse, especially for sex-selective abortion, in January 2008, state drug authorities penalized four chemists for not adhering to prescription norms. As a result, most chemists stopped stocking medical abortion drugs thereby hampering availability in the districts. In October 2008, the state health directorate issued guidelines to districts to facilitate MTP certification of private facilities
Selection and training of ASHAs (accredited social health activists) at the rate of one per village Selection of 42,592 ASHAs approximately one per 1,000 population), who would receive incentives under the JSY for accompanying women for institutional deliveries	So far, 37,431 ASHAs have been recruited. Drugkits have been given to 23,443 ASHAs (43). Training is ongoing in phases. ASHAs have started accompanying women for antenatal check-ups and to institutions for delivery but the proportion of women accompanied compared to total deliveries is low (30)

ANC=Antenatal care; ANM=Auxiliary Nurse Midwife; CHC=Community Health Centre; EmOC=Emergency Obstetric Care; FRUs=First Referral Units; LHV=Lady Health Visitor; MTPs=Medical termination of pregnancies; NGO=Non-governmental organization; NMBS=National Maternity Benefit Scheme; NRHM=National Rural Health Mission; PHC=Primary Health Centre; RCH=Reproductive and child health; SBA=Skilled birth attendance; SCs=Subcentres

An important strategy of the NRHM is the JSY that aims to reduce maternal and infant mortality through promotion of institutional delivery in government health facilities. In Rajasthan, a cash incentive of Rs 1,400 (∼US$ 29) and Rs 1,000 (US$ 21) to each rural and urban woman respectively, was taken to scale in 2006. Over time, the possession of a JSY mother and child card (signaling pregnancy registration and antenatal care) was made mandatory and cheque payments replaced cash transfers. As stated earlier, after JSY, there was dramatic increase in the number of institutional deliveries in Rajasthan (Fig. 3). The monetary benefit to women delivering in facilities has, however, been counteracted by the prevalent system of informal fee collection from families, as indicated in a study comparing home and institutional deliveries and their costs, in rural Rajasthan in 2006-2007 (18). The survey of 1,947 women who delivered recently revealed that families paid substantial amounts for institutional delivery, and most had to take private loans at high interest rates for this purpose. Similarly, a verbal autopsy study of 31 pregnancy-related deaths, indicated that lack of liquid cash contributed to the deaths of several women (44). Lastly, in a preliminary enquiry of 196 women who underwent institutional delivery in a district of southern Rajasthan, families spent a mean of Rs 960 (US$ 20) in a district hospital, Rs 800 (US$ 17) in a CHC, to Rs 650 (US$ 14) in PHCs, on delivery care (Table 19). The cost included money spent on informal fees to doctors, nurses and cleaners, and for the purchase of drugs (16).

**Table 19.** Percentage of JSY beneficiaries who paid money and mean/median paid by category, 2007 (16)

Category	% paid	Mean (Rs)	Median (range)
Doctor's fee	66	426	500 (50-2000)
Nurse's fee	44	236	200 (40-900)
Sanitation staff-fees	73	86	50 (20-400)
Drugs	87	397	300 (50-1400)
Total	99.5	795	750 (0-3700)

JSY=Janani Suraksha Yojana

Recently, the State Government decided to extend the JSY benefits through accredited rural private facilities. Initial accreditation requirements, however, were in line with those required of FRUs (facilities for caesarean section, anaesthesia, obstetricians, etc.). In February 2008, these guidelines were liberalized. The scheme imposes a ceiling of Rs 500 on private facilities, towards fees chargeable from women. This renders accrediting most private-sector delivery services unviable, given the higher input costs of even a normal delivery.

### Rajasthan Health Systems Development Project

A project supported by the World Bank has provided support to the Government of Rajasthan from 2004 to 2009. Project interventions include upgrading district hospitals and health centres, training of staff, improving the quality of clinical services, and strengthening referral systems. Inputs from the project have been deployed towards some of the interventions mentioned in Table 18.

## DISCUSSION

According to direct estimates from the Registrar General of India study, the MMR in Rajasthan has declined. However, the level remains high at 445. Within Rajasthan, little information is available on maternal deaths—the numbers, the causes, and where they occur. While some districts attempted pilot verbal autopsy inquiries of maternal deaths, this was not implemented statewide. The recent increase in deliveries in health facilities could serve as a starting point for introducing facility-based review of maternal deaths in parallel with verbal autopsy of home-level deaths, so as to enhance institutional accountability and to guide programmatic responses.

Till the launch of the CSS programme, maternal health strategies in Rajasthan essentially concentrated on family planning (mainly sterilization) and antenatal care. Only in the late nineties; did emergency obstetric care receive greater investment while skilled birth attendance received attention only after the turn of the century. Our review indicates that, at this point, the state's focus on skilled birth attendance, referral support and EOC are on track but implementation in the districts remains weak. While we have not carried out analysis of financial adequacy of various schemes, under-use of resources for several activities points to the need to strengthen the management capacity of the district. The recent move by the NRHM to appoint district and block programme managers is expected to help address this lacuna. However, district and block managers will need both a strong orientation on effective maternal health strategies and operational autonomy to implement them.

The revised maternity benefit scheme (JSY) has contributed to a large increase in the proportion of institutional deliveries. Although several hitherto dysfunctional CHCs and PHCs started providing delivery services and deliveries at larger hospitals have increased, the quality of care has suffered, with potentially adverse consequences for the woman and foetus. Another concern is early discharge after delivery. If the desired goal of the scheme, i.e. reduction of maternal mortality, is to be achieved, monitoring of not only the numbers but also the quality of services should get attention. Lastly, although services are meant to be free, families continue to pay substantial amounts towards informal fees, transport, medicines, and laboratory tests (16).

By itself, the recent increase in institutional deliveries might not reduce maternal deaths or morbidity. There is a need for concurrent actions to develop a strong referral system and emergency obstetric units. With an increase in the number of institutional deliveries, busy hospitals that already were shouldering a large burden of maternal-foetal complications have had to cope with further increases in routine delivery caseloads. With an inadequate increase in staff and infrastructure to meet this additional load, quality of care is likely to offer. We, therefore, recommend that facilities be decongested through proper use of the primary health-system chain of institutions where the PHCs and CHCs conduct routine deliveries, and only difficult cases be referred to district hospital, or medical colleges. A differential rate of incentive could be given under the JSY with less money offered to women coming straight to district hospitals and medical colleges and more given to those delivering at peripheral institutions. More rural private facilities should also be accredited under the JSY, and the criteria for such accreditation should be similar to those for the government facilities. This would help reduce an overload of patients, thereby maintaining the quality at the government facilities. Clinical audits and case discussions even in the non-teaching hospitals, such as CHC and district hospitals, should be prioritized. Only 15% of the CHCs and 26% of the FRUs in Rajasthan currently have linkages with blood-banks. Since haemorrhage is a leading cause of death, making blood available at facilities conducting large numbers of institutional deliveries should be prioritized by developing blood-storage and transfusion units at the subdistrict level.

Septic abortions contribute greatly to the toll of maternal death. It is, therefore, necessary that larger numbers of care providers are trained in safer techniques of abortion, such as MVA and medical methods. A conscious effort by the Government to improve the number of trained care providers and certified facilities would help reduce deaths and morbidity due to unsafe abortion.

Our review further shows that human-resource capacity, especially of specialists and skilled midwives, has been deficient, and referral arrangements continue to be weak. Non-residence on part of field staff, such as ANMs, whose personal mobility, security, and family needs have not been met, seriously impedes access to round-the-clock services. There is a lack of doctors in the PHCs, especially in tribal districts, and the availability of specialists at higher levels is even worse. Efforts, such as raising salaries or contracting private practitioners, have failed to boost the availability of specialists adequately. The reasons for lack of staff are multiple. While anecdotal evidence points to the apparent perception of lack of safety, especially for female staff in some areas, there is little to attract specialists to government service. Several specialists posted at the CHCs manage to get themselves posted in peri-urban CHCs or ‘on-deputation' in district hospitals. Given the unwillingness of specialists to provide services at rural CHCs, the Government should train and empower much greater numbers of graduate doctors to provide EmOC services. Functions of a comparative EmOC facility can be split. While skills required to carry out caesarean section are much higher and difficult to teach a graduate doctor, skills required to provide blood transfusion can be easily imparted to them. Given that haemorrhage and anaemia are responsible for nearly half of all maternal deaths, ensuring blood-transfusion facilities in the CHCs even when obstetricians are not available (hence, caesarean section not available) would likely make an impact on maternal mortality.

While recruitment of specialists has been difficult at best, there has been a large recruitment drive for staff of SCs. The required ANMs have been posted, and a second ANM has been appointed in each tribal district. However, the ANMs have been largely working as family-planning and immunization workers over the last several decades, and a very few SCs conduct deliveries. Current pre-service training of ANMs does not equip them to function as skilled birth attendants, nor do most of them stay in their field areas. We recommend that the state identifies selected SCs where nurse-midwives would be encouraged to conduct deliveries and manage maternal-neonatal conditions. The in-service training of ANMs in skilled birth attendance that started in several districts over the last year is an encouraging first step. Pre-service training of ANMs also needs to be improved urgently, and their working and living conditions made conducive to staying at the SC. However, if training is to make an impact on performance, careful monitoring of quality of training and post-training performance will be needed, and good performance will need to be rewarded. Further, selected rural facilities need to be strengthened to impart skill-based training in birth attendance and EmOC. An integrated training plan at the state level should be made so that training efforts under the various national health programmes are coordinated rather than remaining as discrete activities. Because medical colleges impart training in an over-medicalized manner, skill-based training of primary-care staff should remain at primary-level institutions, such as CHCs but with improved quality of clinical care. Even after training in skilled attendance, nurse-midwives working at most CHCs and PHCs do not conduct deliveries or manage women with obstetric complications. This is partly related to the informal fees levied for conducting delivery and partly due to lack of clarity on the part of doctors in-charge, who continue to believe that only doctors can provide services and that nurse-midwives can only ‘assist' them. Even when doctors are on leave, nurses-midwives at such facilities might turn back women coming with labour for fear they will be responsible, should something go wrong. Hence, it is important that the Government issues appropriate guidelines for PHCs and CHCs authorizing and directing nurse-midwives to provide maternal services in accordance with the Government of India guidelines.

## ACKNOWLEDGEMENTS

The study was financially supported by John D. and Catherine T. MacArthur Foundation, New Delhi and Chicago and by the Department for International Development (DfID), UK, through ICDDR,B, Dhaka, Bangladesh and Indian Institute of Management, Ahmedabad. The funders had no involvement in the research, writing, or in the decision to submit the paper for publication.
